# Supplementary material for: TastepepAI: An artificial intelligence platform for taste peptide de novo design
Source: PLoS Comput Biol. 2025 Oct 16;21(10):e1013602. doi: 10.1371/journal.pcbi.1013602 (PMC12543283; doi:10.1371/journal.pcbi.1013602)
Supplement: S1 File — (ZIP) [file pcbi.1013602.s006.zip › Supplementary Data 2/TaPep16 HPLC.doc]

**HPLC REPORT**

| Sample: | Fcl-NJP93317 TaPep16 FD-6 | | | Analyzed date: | 2024-9-23 |
| --- | --- | --- | --- | --- | --- |
| Analyst: | LY Reconstitution: 1mg/0.5ml H_2_O | | | | |
| Lot. No.: | P240913-MX1172232 | | | | |
| Column: | 250*4.6mm,ChromCore 120 C18 5um | | | | |
| Solvent A | A: 0.1% Trifluoroacetic Acid in 100% Acetonitrile | | | | |
| Solvent B | B: 0.1% Trifluoroacetic Acid in 100% Water | | | | |
| Gradient: |  | A | B |  | |
|  | 0.0min | 10% | 90% |  | |
|  | 25.0min | 35% | 65% |  | |
|  | 25.1min | 100% | 0% |  | |
|  | 30.0min | Stop | |  | |
| Volume: | 10μl | | |  | |
| Wavelength: | 220nm | | |  | |
| Flow rate: | 1.0ml/min | | |  | |
| ───────────────────────────────────────────────────  Peak No Ret Time Height Area Conc.  ───────────────────────────────────────────────────  1 9.447 575.158 5984.445 0.0503  2 9.982 1217.286 8344.901 0.0701  3 10.892 2890.667 39772.535 0.3343  4 11.330 733392.500 11458611.000 96.3095  5 11.832 13948.783 169198.328 1.4221  6 12.138 5569.381 67136.180 0.5643  7 13.523 988.191 13160.569 0.1106  8 15.300 306.110 2573.954 0.0216  9 16.475 4636.261 53857.793 0.4527  10 17.542 5179.738 57157.168 0.4804  11 21.408 1493.455 14825.552 0.1246  12 22.540 221.690 780.102 0.0066  13 23.118 836.512 6295.913 0.0529  ───────────────────────────────────────────────────  Total 100.00 | | | | | |
